# Urinary and breast milk biomarkers to assess exposure to naphthalene in pregnant women: an investigation of personal and indoor air sources

**DOI:** 10.1186/1476-069X-13-30

**Published:** 2014-04-27

**Authors:** Amanda J Wheeler, Nina A Dobbin, Marie-Eve Héroux, Mandy Fisher, Liu Sun, Cheryl F Khoury, Russ Hauser, Mark Walker, Tim Ramsay, Jean-François Bienvenu, Alain LeBlanc, Éric Daigle, Eric Gaudreau, Patrick Belanger, Mark Feeley, Pierre Ayotte, Tye E Arbuckle

**Affiliations:** 1 Water and Air Quality Bureau, HECSB, Health Canada, 269 Laurier Avenue West, AL 4903C, Ottawa, ON K1A 0K9, Canada; 2 Environmental Health Science and Research Bureau, Health Canada, 50 Colombine Dr., AL 0801A, Ottawa, ON K1A 0K9, Canada; 3 Department of Environmental Health, Harvard School of Public Health, Boston, MA 02115, USA; 4 Ottawa Hospital Research Institute, University of Ottawa, 501 Smyth Road, Ottawa, ON K1H 8L6, Canada; 5 Centre de toxicologie du Québec, Institut national de santé publique du Québec (INSPQ), Québec, Canada; 6 Bureau of Chemical Safety, Health Canada, Ottawa, ON K1A 0K9, Canada; 7 Axe santé des population et pratiques optimales en santé, Centre de recherche du CHU de Québec and Université Laval, Québec, Canada; 8 Currently affiliated with the World Health Organization European Centre for Environment and Health, Platz der Vereinten Nationen 1, Bonn 53113, Germany; 9 Centre for Ecosystem Management, School of Natural Sciences, Edith Cowan University, 270 Joondalup Drive, Joondalup, WA 6027, Australia

**Keywords:** Naphthalene, Personal exposure, Biomarkers, Indoor air quality

## Abstract

**Background:**

Naphthalene exposures for most non-occupationally exposed individuals occur primarily indoors at home. Residential indoor sources include pest control products (specifically moth balls), incomplete combustion such as cigarette smoke, woodstoves and cooking, some consumer and building products, and emissions from gasoline sources found in attached garages. The study aim was to assess naphthalene exposure in pregnant women from Canada, using air measurements and biomarkers of exposure.

**Methods:**

Pregnant women residing in Ottawa, Ontario completed personal and indoor air sampling, and questionnaires. During pregnancy, pooled urine voids were collected over two 24-hour periods on a weekday and a weekend day. At 2–3 months post-birth, they provided a spot urine sample and a breast milk sample following the 24-hour air monitoring. Urines were analyzed for 1-naphthol and 2-naphthol and breast milk for naphthalene. Simple linear regression models examined associations between known naphthalene sources, air and biomarker samples.

**Results:**

Study recruitment rate was 11.2% resulting in 80 eligible women being included. Weekday and weekend samples were highly correlated for both personal (r = 0.83, *p* < 0.0001) and indoor air naphthalene (r = 0.91, *p* < 0.0001). Urine specific gravity (SG)-adjusted 2-naphthol concentrations collected on weekdays and weekends (r = 0.78, *p* < 0.001), and between pregnancy and postpartum samples (r = 0.54, *p* < 0.001) were correlated.

Indoor and personal air naphthalene concentrations were significantly higher post-birth than during pregnancy (*p* < 0.0001 for signed rank tests); concurrent urine samples were not significantly different. Naphthalene in breast milk was associated with urinary 1-naphthol: a 10% increase in 1-naphthol was associated with a 1.6% increase in breast milk naphthalene (95% CI: 0.2%-3.1%). No significant associations were observed between naphthalene sources reported in self-administered questionnaires and the air or biomarker concentrations.

**Conclusions:**

Median urinary concentrations of naphthalene metabolites tended to be similar to (1-naphthol) or lower (2-naphthol) than those reported in a Canadian survey of women of reproductive age. Only urinary 1-naphthol and naphthalene in breast milk were associated. Potential reasons for the lack of other associations include a lack of sources, varying biotransformation rates and behavioural differences over time.

## Background

Naphthalene is an abundant polycyclic aromatic hydrocarbon (PAH) found in urban environments. It is typically present in the gas-phase under usual ambient conditions and is routinely detected in both indoor and outdoor environments. Most naphthalene exposures for non-occupationally exposed individuals occur primarily indoors at home [1]. US ATSDR considers naphthalene as reasonably anticipated to be a human carcinogen [2] while IARC has classified naphthalene as a possible human carcinogen (Group 2B) [3]. Health Canada’s long-term (≥24-hour) maximum exposure limit for residential naphthalene indoor air concentrations is 10 μg/m^3^[4].

There are a number of known naphthalene indoor sources and these include pest control products, (i.e., moth balls), incomplete combustion such as cigarette smoke, woodstoves and cooking, as well as some consumer and building products [1,5-7]. Indoor naphthalene concentrations have also been shown to be elevated in homes with smokers versus homes without smokers [8,9] and can off-gas and volatilize from vehicles and stored petroleum products found in attached garages [9-11].

Outdoor sources of naphthalene include exhaust from vehicles, including diesel and gas-powered equipment, as well as vapours from petroleum products. Other sources that are less common include asphalt, forest fires and some industrial processes [9,12,13].

To understand total human exposure to naphthalene a limited number of studies have included both personal air sampling along with biomonitoring [14,15]. Uptake, absorption, distribution, and metabolism can be affected by individual physiological differences and behaviours [14]. The overall rate of metabolism of naphthalene by humans is unknown, although it has been suggested that there is a two-phase excretion of 1-naphthol in urine. The first phase exhibits a half-life of approximately 1.2 – 1.9 hours while the second phase is 14 – 46 hours [16]. Naphthalene is metabolically activated by forms of cytochrome P450 to naphthalene 1,2-oxide, which can be detoxified by glutathione-Ѕ-transferase (GST) to eventually be excreted as mercapturic acids in urine or spontaneously convert to 1- and 2-naphthol and be eliminated in the urine as glucuronides and sulfates. Naphthalene 1,2-oxide can also undergo other transformations to dihydroxydimethylthio and trihydroxymethylthio metabolites and trihydroxymercapturic acid in urine [17].

Biomonitoring can provide insight into the uptake of naphthalene. Only a few studies [18-20] have attempted to measure naphthalene in breast milk and to date, there are no Canadian data available. Conjugates of 1-naphthol and 2-naphthol in urine have been associated with predicted concentrations in the breathing zone but there are limited data available in non-occupationally exposed individuals [14]. Meeker et al. [21] recommended that the ratio of 1-naphthol to 2-naphthol be used to identify the metabolism of naphthalene. They identified situations where discrepancies between concentrations of these metabolites (e.g., ratio >2) were in fact related to the metabolism of the insecticide carbaryl (1-naphthyl methylcarbamate) which is primarily excreted as 1-naphthol.

As pregnancy is associated with a number of physiological changes in women that could affect the toxicokinetics of chemicals [22] and there are critical periods of development during pregnancy for the fetus, it is important to study the exposure of pregnant women to potentially harmful chemicals such as naphthalene. Reports of probable fetal exposure after maternal inhalation or ingestion of naphthalene have been documented in the scientific literature [22,23]. Infants, particularly those with a glucose-6-phosphate dehydrogenase (G6PD) deficiency, may be particularly sensitive to naphthalene exposure. Cases of hemolytic anemia, sometimes leading to more serious outcomes (e.g., kernicterus (irreversible neurological impairment) and death), have been reported in infants exposed to naphthalene-treated household items [24-32]. Authors of a recently published New York study of 5-year old children’s urinary naphthalene metabolite concentrations identified an association with chromosomal aberrations (including translocations) which are precancerous changes in adults [33].

We conducted a cohort study in a group of pregnant women residing in Ottawa, Ontario, Canada to assess naphthalene exposure and biomarkers in maternal urine and breast milk. We assessed naphthalene sources and concentrations inside residences, along with personal exposure measures, to assist in determining both source and route-specific information related to naphthalene exposure in a non-occupationally exposed population. This study addresses current knowledge gaps by attempting to measure naphthalene body burdens, identify major sources of naphthalene exposure, and quantify their contribution to an individual’s exposure.

## Methods

Pregnant women (<20 weeks gestation) from the Ottawa area were recruited to participate in the P4 Study: Plastics and Personal-care Product Use in Pregnancy, a wider study investigating pregnant women’s exposure to a range of chemicals. This manuscript focusses on the personal and indoor air exposure to naphthalene and resulting biomarkers. Recruitment was clinic-based and occurred at an obstetrical clinic at The Ottawa Hospital (TOH) and a privately run obstetrical clinic. Posters and pamphlets about the study were placed in the obstetrical and ultrasound clinics of TOH and physician offices. Research nurses from the clinical sites were trained in patient screening, recruitment, obtaining consent, specimen and data collection, and processing, as well as the shipment of biospecimens.

The women completed detailed consumer product diaries along with noting any use of products containing naphthalene for a 48-hour period during the early pregnancy visits and 24-hours prior to the post-partum visit. Typically the biomarker and air collection started at the midpoint of the diary, i.e. 24-hours after the start of the diary.

### Biomarker collection and analysis

For the purposes of this analysis, the women provided urine samples on three occasions: twice during pregnancy (<20 weeks) and once at two to three months post-birth. In order to assess activity related differences in exposure, prior to 20 weeks of pregnancy women were asked to collect all voids over two 24-hour periods (multiple spot urines) – once on a week day and again on a weekend day. After collection, a small equal amount from each void during the 24-hour period was pooled to create an aggregated sample for naphthalene biomarker analysis. At two to three months post-birth, the women provided a single spot urine sample at the end of the 24-hour air monitoring period. These samples were stored at -20°C until analyses. One paper has reported that 1- and 2-naphthol in urine was stable for at least 1 month at -20°C [34].

Analyses of 1-naphthol and 2-naphthol were undertaken on the 24-hour aggregated pregnancy urine samples and the post-birth spot sample using the following method. Internal standards (1-naphthol-d_7_ and 2-naphthol-d_7_) were added to a 1 mL volume of urine. The conjugated forms of 1-naphthol and 2-naphthol were hydrolyzed at 37°C for 16 hours with β-glucuronidase (*helix pomatia*). The extraction of the analytes was performed on a mixed mode solid-phase extraction (SPE) cartridge Oasis MAX (Waters), 60 mg. The analytes were eluted with methanol, dried and reconstituted in 200 μL of a mixture of mobile phase A and B (72:28) containing gallic acid (200 mg/L). The extracts were analyzed by UPLC-MS-MS (Acquity UPLC system and Xevo TQ-S tandem mass spectrometer, Waters; Milford, MA). The LC separation was performed on a Halo C18 column (2.1 × 50 mm, 2.7 μm, Advanced Materials Technology) with 0.01% NH_4_OH in water as mobile phase A and acetonitrile as mobile phase B. The separation was achieved isocratically with 28% of B over 3.5 minutes, and the column was flushed with 100% B for 0.3 minutes at a flow rate of 0.5 mL/min. The total run time was 4 minutes. The limits of detection for 1-naphthol and 2-naphthol were 0.03 μg/L, and the calibration curves were linear up to 50 μg/L. Field blanks for the urinary samples were analyzed and no contamination was found. The analytes were monitored by Multiple Reaction Monitoring (MRM) in the negative mode for the following ions: 1-naphthol and 2-naphthol : m/z 143.0 > 115.1 (quantifier) and 143.0 > 143.0 (qualifier)1-naphthol-d_7_ and 2-naphthol-d_7_ : m/z 150.0 > 122.1 (quantifier) and 150.0 > 150.0 (qualifier).

Quality control (QC) materials, including method analytical blanks, were prepared from human urine obtained from volunteers in the analytical laboratory. The urine, previously tested for 1- and 2-naphthol content, was spiked, with a solution of 1- and 2-naphthol from a different supplier, at a concentration of 20 μg/L to obtain a high concentration QC material. The low concentration QC material was composed of the unchanged urine (concentrations: 1.0 μg/L for 1-naphthol and 0.5 μg/L for 2-naphthol). The two QC materials were used in alternation and placed after each set of ten samples in each analytical batch. The INSPQ laboratory participates in the German external quality assessment scheme (http://www.g-equas.de/) in which 1- and 2-naphthol are assayed.

As concentrations derived from urine may be affected by the dilution of the urine, concentrations were corrected by the specific gravity (SG) of the sample. The following formula was used (adapted from Just et al., [35]):

P_c_ = P_i_ [(SG_m_– 1)/(SG_i_ – 1)], where *P*_*c*_ is the specific gravity-adjusted metabolite concentration (ng /mL), *P*_i_ is the observed metabolite concentration, and *SG*_i_ is the specific gravity of the urine sample and SG_m_ is the median specific gravity for the cohort.

Breast milk was collected at the two to three month post-birth visit, at the end of the 24-hour air monitoring period. The breast milk sample was collected by either hand or pump in a glass container, kept cool until delivered to the laboratory where it was transferred to 30 mL Nalgene® containers and stored at -20°C until analysis as per methods described by other studies [19,20]. Breast milk was analyzed for naphthalene using the following method. Briefly, the internal standard (naphthalene-d_8_) was added to a 1 mL volume of breast milk. The extraction of naphthalene was performed with a silicone/PFTE septum, by heating at 80°C for 16 hours. The septum was transferred into a headspace vial, incubated at 145°C for 5 minutes and injected by the headspace technique on a GC-MS-MS (7890A gas chromatograph with 7000B tandem mass spectrometer, Agilent Technologies; Mississauga, Ontario, Canada) equipped with a PAL Combi-xt injector (Leap Technologies; Carrboro, NC, USA). The GC separation was achieved on a DB-5 ms column (30 m × 0.25 mm × 0.25 μm, Agilent Technologies). The temperature of the injector was 250°C and the temperature gradient was: Initial temperature of 100°C for 0.5 minutes, then 40°C/minute until 320°C, then hold for 2 minutes. Carrier gas was helium at a flow rate of 2 mL/min. The limit of detection for naphthalene was 0.03 μg/L. The analytes were monitored by Multiple Reaction Monitoring (MRM) in the positive mode for the following ions:naphthalene : m/z 128 > 128 (quantifier) and 128 > 102 (qualifier)naphthalene-d_8_ : m/z 136 > 136 (quantifier) and 136 > 108 (qualifier).

Quality control (QC) materials, including method analytical blanks, were prepared from human milk obtained from volunteers in the analytical laboratory. The milk, previously tested for naphthalene content, was spiked with a solution of naphthalene from a different supplier, at a concentration of 10 μg/L to obtain a high concentration QC material, and a concentration of 0.4 μg/L to obtain a 0.5 μg/L low concentration QC material. The two QC materials were used in alternation and placed after each set of ten samples in each analytical batch. While no studies on the stability of naphthalene in milk stored at -20°C could be found in the literature, one study has reported naphthalene contamination from packaging materials of milk samples stored at room temperature in low-density polyethylene containers [36]. However, our breast milk was collected in glass jars, kept refrigerated until aliquoted in the laboratory into Nalgene® containers and immediately frozen at -20°C. The Mendela® breast pump provided to participants was tested and no naphthalene was detected.

As naphthalene is fat soluble and concentrations are affected by the lipid concentrations in an individual’s breast milk, the naphthalene concentrations were corrected for lipid concentration and are reported in ng/g lipid.

### Air monitoring and analysis

Personal and indoor air measures of naphthalene were completed concurrently with the 24-hour urine collection and prior to the spot urine and breast milk collection. Personal air monitoring was completed in the women’s breathing zone by attaching the sampler to their collar, while indoor air monitoring required the women to place a sampler in their living rooms at a height of approximately 1.5 m, away from any sources of heat. Each passive sampler measured 24-hour air samples for naphthalene (OVM 3500, 3 M, St. Paul, MN). As the air monitoring was participant-based, replicate sampling was not attempted due to the complexity of conducting this additional monitoring.

Naphthalene was extracted using toluene, which was previously demonstrated to have a recovery of 72%. The analysis protocol has been described previously [37]. Briefly, this involved extracting the samples with 2 mL of toluene for one hour on a mechanical shaker. The toluene extraction solvent was spiked with 1,2-dichlorobenzene-d_4_ (1.34 ng/μL). The extraction solvent was then transferred to a 1.5 mL autosampler vial and analyzed via GC-MS (HP5890 II GC & HP5792 MS). The GC was equipped with a capillary column (J&W 123–1364 DB-624, 60 m × 0.32 mm × 1.8 μm). The carrier gas (helium) head pressure was 6.0 PSI and injector and detector temperatures were kept constant at 220°C and 260°C, respectively. The temperature program yielded a 12.9 min retention time (initial temperature, 80°C for 1 min, 80°C to 260°C at 15°C/min, hold for 1.5 min). The MS was configured to quantify the following 3 characteristic ions of naphthalene: 128, 102 and 64 amu. The ion ratios and peak integration were verified manually for each sample.

The naphthalene concentrations were calculated using the mass adsorbed on each sampler, the specific uptake rate for naphthalene, exposure times to the nearest one minute and laboratory blank PSDs analyzed at the same time as the samples. The method detection limit (MDL), including handling and extraction was determined by the CFR 40 method. The MDL was 0.1 μg/m^3^[37].

To calculate concentrations, the laboratory results were merged with log sheet data. Concentrations were calculated based on sample mass, sampling duration, sampling rate and recovery efficiency. All samples were coded as valid, flagged, or invalid, based on the sampling period and field technician comments. Samples with a sampling period ± 25% of the target duration (24-hours) were deemed invalid. If the sampling period was ± 12.5 to 25%, the samples were flagged. Samples with technician comments such as container not sealed on time, unknown sampling location, were also flagged.

### Statistical methods

Given the naphthalene exposures were not normally distributed, Spearman correlations were conducted to determine correlations between the air and urine naphthalene measurements within visits and across visits. The non-parametric signed rank test was used to test differences between levels measured at different visits. Intra-class correlation coefficients (ICC) were calculated using a one way random effects model (Proc Mixed) on air and biomarker concentrations that were transformed using the natural logarithm. ICC measures the ratio of between-subject variance to total variance ranging from 0 (meaning no within person reproducibility) to 1 (meaning perfect reproducibility). We defined 0.75 as high; 0.40 to 0.75 as moderate; below 0.40 as poor reproducibility [38]. Simple linear regression models were used to examine associations between a number of known naphthalene sources and measured concentrations in log transformed air and biomarker samples. As there were no significant differences between air or urinary biomarker concentrations measured on the weekday and weekend pregnancy visit, these were averaged for the pregnancy models; separate models were created for the pregnancy and the post-partum visits.

The independent variables examined were: age, body mass index (BMI) (calculated from pre-pregnancy weight), season, moth ball use, exposure to smoke (current smoker, previously a smoker, exposure to second hand smoke (SHS) and exposure to SHS in the home), exposure to traffic pollutants including the presence of an attached garage, density of roads and highways in neighbourhood (total road or expressway segment length in a participant’s 3-digit postal code divided by the area of the 3-digit postal code), exposure to indoor combustion (presence of wood-burning fireplace), and type of ventilation and heating in the home. Naphthalene in indoor and personal air, 1-naphthol and 2-naphthol in urine, and naphthalene in breast milk were examined as the dependent variables in turn. In biomarker models, personal and indoor air naphthalene concentrations were also entered as independent predictors in turn.

Since 1-naphthol in urine can also originate from exposure to carbaryl, we examined the ratio of 1-naphthol to 2-naphthol as an indicator of its source. Ratios above 2 may indicate that a portion of the 1-naphthol originated from carbaryl rather than naphthalene exposure [21,39]. Where this occurred, a sensitivity analysis excluding these individuals was conducted. All data processing and analyses were conducted using SAS Enterprise Guide 4.2 (SAS Institute, Inc.).

## Results

A total of 1307 potential research participants were approached to enter the study from November 2009 to December 2010. 769 potential participants were eligible for the study of whom 86 were recruited during this time period, with an acceptance rate of 11.2%. The reasons for the low recruitment included significant participant burden, no interest in participating, too busy, and unease about wearing the air monitors in public. There were a total of 86 participants recruited, six participants agreed to participate and signed the consent form but then shortly afterwards withdrew leaving 80 participants who completed the first visit in early pregnancy. 70 participants completed visit 2, 71 completed visit 3, 73 had completed chart reviews at delivery and 63 completed the final post-partum visit. A total of 7 participants withdrew from the study, 7 were lost to follow-up and three had early outcomes (miscarriage, stillbirth, neonatal death). Initially recruitment of the women was aimed at before the 14^th^ week of pregnancy; however, this had to be expanded to include the window of 19 weeks 6 days gestation in the winter of 2010 due to low recruitment as women seemed hesitant to participate early in pregnancy. This change dramatically increased recruitment.

Table 1 includes details of the participant characteristics. The average age of participants at time of delivery was 33 years, with a range from 20 to 47 years. This was the first pregnancy for 37 of the participants. Fifty-five percent of the participants had a household income exceeding $100,000CDN and 89% had a college or university degree.

**Table 1:** Characteristics of the study population

Participant characteristics and exposures*		**Frequency (%) or mean (SD) (N = 80)**	
Age (years)		32.4 ± 5.0	
BMI (Kg/m^2^)		24.0 ± 4.3	
Education level – college/university degree		71 (89)	
Household income	Below $50 k	4 (5)	
	Above $100 k	44 (55)	
Parity	Primiparous	37 (46)	
	Multiparous	43 (54)	
Ever smoked more than 100 cigarettes		25 (32)	
Smoke currently		2 (3)	
Exposed to second hand smoke		18 (23)	
Exposed to second hand smoke inside home		3 (4)	
Road density in postal code (km/km^2^)		2.03 ± 1.32	
Highway density in postal code (km/km^2^)		0.41 ± 0.37	
**Home Characteristics**^**#**^		**Pregnancy (N = 56)**	**Post-birth (N = 53)**
Moth balls used		5 (9%)	0 (0%)
House with an attached garage and connecting door		25 (45%)	24 (45%)
Fireplace		33 (59%)	33 (62%)
Wood		16 (29%)	13 (25%)
Natural gas		14 (25%)	17 (32%)
Heating type			
Electric		3 (5%)	6 (11%)
Natural gas		46 (82%)	39 (74%)
Oil		4 (7%)	6 (11%)

*Participant characteristics were asked at the pregnancy visit only.

^#^Home characteristic questionnaires were not filled out by all participants in each phase of the study. The total number filled out in each period is given in brackets. Models examining these variables were limited to participants with reported data.

For the air monitoring component, a total of 375 participant days were completed. These included 322 valid samples, 28 flagged samples due to sampling times being 12.5 to 25% greater or less than the targeted 24-hours, 24 invalid samples due to sampling times being beyond the targeted 24-hours ± 25% or due to damage of the sampler, and 1 sample below detection. Blank corrections were not required as no blanks had detectable concentrations of naphthalene. There were no duplicates collected due to the complexity of having participants complete their own data collection.

A total of 191 urine samples (all samples were included from both of the pregnancy visits) were analyzed for 1-napthol and 2-napthol (62 from weekday and 67 from weekend visits during pregnancy, and 62 from the post-delivery visit). Fifty-two breast milk samples were analysed for naphthalene.

Descriptive statistics for the naphthalene in air and breast milk, and the biomarker urinary metabolites are presented in Table 2. Results for the urinary biomarkers are presented with and without adjustment for specific gravity (SG), which accounts for differences in individual hydration status. SG-adjusted geometric mean urinary concentrations tended to be higher than the corresponding unadjusted concentrations. Median SG-adjusted urinary concentrations of 1-naphthol collected on a weekday and weekend day were 1.19 and 1.15 μg/L, respectively. SG-adjusted median concentrations of 2-naphthol in urine varied from 2.53 to 3.33 μg/L, depending on when the sample was collected. Concentrations of SG-adjusted 1- and 2-napthol in urine did not significantly differ between the three collection periods.

**Table 2:** Descriptive statistics of measured naphthalene and metabolite concentrations in environmental and biological samples in the P4 Study

**Sample**	**Pregnancy - weekday**							**Pregnancy - weekend**							**Weekday vs weekend day**	**2-3 months post-birth**							**Pregnancy vs. post-birth**
	**N**	**Q1**	**Median**	**Q3**	**GM (GSD)**	**95**^**th**^**% ile**	**Min – Max**	**N**	**Q1**	**Median**	**Q3**	**GM (GSD)**	**95**^**th**^**% ile**	**Min – Max**	**Sign rank test**	**N**	**Q1**	**Median**	**Q3**	**GM (GSD)**	**95**^**th**^**% ile**	**Min – Max**	**Sign rank test**
**Air (μg/m**^**3**^**)**																							
Personal	56	0.38	0.73	1.03	0.7 (2.18)	3.14	0.2 –6.37	58	0.37	0.79	1.21	0.79 (2.41)	3.9	0.2 - 14.85	0.10	61	1.09	1.74	2.46	1.68 (1.86)	4.51	0.3 - 12.31	<0.0001
Indoor	57	0.5	0.68	1.06	0.8 (2.01)	3.51	0.27 –5.97	58	0.45	0.73	1.05	0.76 (2.05)	3.56	0.22 -4.79	0.55	60	1.14	1.83	2.48	1.79 (1.82)	4.94	0.31 - 11.71	<0.0001
**Urine (μg/L)**																							
1-naphthol	62	0.73	1.14	2.28	1.32 (2.80)	6.06	0.13 - 126.08	67	0.67	1.05	1.91	1.16 (2.44)	3.85	0.23 - 81.57		62	0.65	1.06	1.62	1.04 (2.69)	6.16	0.14 - 11.62	
1-naphthol – SG* corrected	62	0.7	1.19	2.22	1.34 (2.72)	6.3	0.2 - 140.7	67	0.78	1.15	1.74	1.23 (2.41)	3.68	0.22 -133.4	0.34	62	0.73	1.09	1.67	1.23 (2.08)	4.46	0.38 - 12.48	0.4
2-naphthol	62	1.7	2.73	5.09	2.92 (2.08)	9.57	0.81 -16.41	67	1.72	2.56	4.29	2.72 (2.14)	12.4	0.68 - 19.93		62	1.56	2.86	5.85	2.86 (2.86)	13.47	0.19 - 32.8	
2-naphthol – SG* corrected	62	1.73	2.53	4.7	2.94 (1.99)	9.47	0.87 - 14.43	67	1.97	2.84	3.95	2.92 (1.93)	11.56	0.77 - 17.53	0.44	62	2.09	3.33	5.66	3.39 (2.16)	12.19	0.74 - 25.39	0.15
**Breast Milk (ng/g lipid)**																							
Naphthalene															N/A	52	6.06	7.55	13.05	9.12 (1.92)	40.17	3.86 – 79.36	N/A

*Specific gravity.

Indoor and personal air naphthalene concentrations did not significantly differ between the weekday and weekend visits (Table 2). However, both indoor and personal air measurements were significantly higher at the post-partum visit than at the pregnancy visits (*p* < 0.0001 for signed rank tests). The air samples were analysed throughout the course of the data collection with some samples from the pregnancy visits and post-partum visit being analysed at the same time, therefore reducing the likelihood of bias resulting from differences in timing of analyses.

Personal and indoor air naphthalene concentrations collected during pregnancy were highly correlated, within each visit and across visits with Spearman Correlation Coefficients ranging from 0.83 to 0.91 (*p*-value = <.0001). The average of the two pregnancy measures and the postpartum concentrations were also significantly correlated, personal r = 0.4 (*p*-value = 0.0026) and indoor r = 0.46 (*p*-value = 0.0004).

Significant correlations were observed in the urinary SG-adjusted 2-naphthol concentration in samples collected during pregnancy on weekdays and the weekend (r = 0.78, *p* < 0.001) and when comparing pregnancy and postpartum samples (r = 0.54, *p* < 0.001). There were no significant correlations seen with the postpartum breast milk samples with any of the air or urine measures.

The ICC analysis suggested moderate reproducibility for SG-adjusted 2-naphthol across the study period (weekday, weekend and post-partum samples) (ICC = 0.66). The ICCs were low for indoor air (ICC = 0.31), personal air (0.32) and 1-naphthol (0.24).

Simple linear regression models examined associations between various naphthalene sources (e.g., moth ball use, exposure to smoke, attached garage, density of roads and highways in neighbourhood, presence of a wood-burning fireplace, type of ventilation and heating in the home) and measured concentrations of naphthalene in air and biomarkers; no significant associations were found (all *p*-values greater than 0.05, see Additional file 1: Table S1). Similarly, no consistent associations were seen with age or BMI. The data suggested that samples collected during the winter had lower indoor and personal air naphthalene concentrations compared to summer. This was significant at the 0.05 level for indoor air concentrations post-partum, and for personal air concentrations during pregnancy on both sampling days (see Table 3). The weekday 2-napthol concentrations were significantly different in the fall and winter compared to the summer. We did observe a small association between urinary 1-naphthol concentrations and naphthalene in breast milk despite there being no correlations: a 10% increase in 1-naphthol in urine was associated with a 1.6% increase in naphthalene in breast milk (95% CI: 0.2% - 3.1%). Two observations were removed from this analysis due to their high influence on the association, as indicated by Cook’s distance.

**Table 3:** **Seasonal simple linear regression results for naphthalene in air (μg/m**^**3**^**)**

**Exposure**	**Season**	**N**	**Median**	**Mean**	**GM**	*p*-value*
Weekday Indoor	Fall	19	0.947	1.254	1.029	0.341
	Spring	13	0.827	1.349	0.903	0.728
	Summer	15	0.780	1.005	0.827	
	Winter	15	0.489	0.626	0.524	0.063
Post-Partum Indoor	Fall	9	1.024	1.532	1.258	0.006
	Spring	22	1.798	1.923	1.815	0.074
	Summer	8	2.355	3.164	2.781	
	Winter	19	1.849	2.223	1.639	0.031
Weekday Personal	Fall	20	0.950	1.423	1.083	0.583
	Spring	13	0.801	1.156	0.840	0.695
	Summer	15	0.734	1.449	0.940	
	Winter	13	0.295	0.477	0.338	0.001
Post-Partum Personal	Fall	9	1.023	1.183	1.153	0.002
	Spring	22	1.739	1.742	1.665	0.031
	Summer	8	2.501	3.219	2.819	
	Winter	20	1.556	2.236	1.538	0.015

*Simple Linear Regression with Summer as the reference category.

There were 4 women in the pregnancy period and 2 women in the post-birth period with 1-naphthol to 2-naphthol ratios above 2 which could be indicative of exposure to carbaryl as opposed to naphthalene. Excluding these individuals from the simple linear regression models did not change the results, with the exception of the association between urinary 1-naphthol and naphthalene in breast milk; this association was reduced to a 1.1% (95% CI: -0.3% - 2.6%) increase in naphthalene with a 10% increase in 1-naphthol. This association should be interpreted with caution due to the small sample size measured, and the fact that the association was reduced when we removed individuals suspected of having alternative sources of 1-naphthol.

## Discussion

The measured indoor air concentrations of naphthalene (medians across the 3 sampling periods ranging from 0.68 to 1.83 μg/m^3^) are comparable to findings from previous studies. Jia and Batterman [9] summarized residential indoor air naphthalene concentrations for studies conducted from 1986 to 2006. The authors reported a median concentration range for homes without smokers of 0.18 to 1.7 μg/m^3^. Only one indoor air measurement from the three monitoring sessions in this study was found to be above the Health Canada guideline of 10 μg/m^3^[4] at 11.71 μg/m^3^. This measurement was taken at the post-birth visit; the source for this higher level was not clear but the pregnancy-period measurement at the same home was well below the guideline (3.03 μg/m^3^) suggesting that this was not the result of an ongoing source.

Canadian levels of indoor air naphthalene have been measured in several studies. The median naphthalene level in Quebec City homes without smokers was 1.12 μg/m^3^ for a seven day integrated sample [40]. Measurements made in a population-based study of Canadian homes in 1991 over multiple seasons had a range of 24-hour mean concentrations of 1.10 – 8.10 μg/m^3^[41]. An Ottawa based study of 75 residences had a 24-hour mean of 3.87 μg/m^3^. These studies included both homes with and without smokers [42]. Indoor air naphthalene was also measured in homes without smokers in Edmonton, Alberta, where 7-day median concentrations were 0.32 and 0.29 μg/m^3^, for winter and summer seasons respectively [43]. This contrasts with our findings where summer indoor concentrations were significantly higher than both fall and winter. The sampling time frame for the different studies may explain some of the differences in concentrations of naphthalene in indoor air.

Very little data exists on personal monitoring for naphthalene exposure; however, the concentrations we measured among pregnant women (medians ranging from 0.73 to 1.74 μg/m^3^) are slightly lower than concentrations found in other populations. An Italian study of non-occupationally exposed adults living and working in Milan and the surrounding areas included 108 subjects, 18 of whom completed both personal air sampling and urine samples which were analysed for naphthalene. Median personal naphthalene air samples taken during the 5-hour work period was 3.4 μg/m^3^ (interquartile range: 1.4 – 4.9 μg/m^3^), with no differences between smokers and non-smokers. They also measured concentrations of un-metabolized naphthalene in urine (median: 46 ng/L, interquartile range: 41 – 56 ng/L) but found no associations between the personal air and urine naphthalene concentrations. They were also unable to identify any predictors for either the air or urine samples [44].

In an Atlanta, GA-based study of 8 non-occupationally exposed individuals who completed a personal air sample along with urine samples, median exposure levels to naphthalene ranged from a low of 0.13 μg/m^3^ at work (interquartile range: 0.095–0.22 μg/m^3^) to a high of 0.92 μg/m^3^ indoors at home (interquartile range: 0.37–3.27 μg/m^3^). Indoor home concentrations were higher than concentrations measured while driving, which reflect the importance of residential indoor sources for naphthalene. A comparison of personal air measurements and urinary excretion of naphthalene between days with high and low PAH diets suggested that inhalation is the primary route of exposure for naphthalene [14].

The study by Bouchard et al., [45] asked participants to record habits and activities involving potential PAH exposures. They were only able to identify passive smoking with exposure associated with higher 2-naphthol excretion. The authors felt that the absence of a link between most of the variables from the questionnaire and the urinary excretion of PAH biomarkers was due to the low reporting of exposure to these variables.

Indoor air measurements of naphthalene have previously been recognized to be a good proxy for personal naphthalene air exposures [46]. The high correlations we observed between indoor and personal naphthalene concentrations confirm this, see Additional file 2: Figure S1.

There are a number of sources of naphthalene in the indoor environment, including moth balls and deodorizers, smoking, attached garages, construction and wood products, indoor combustion, and heating systems [9,47]. Although we examined a number of known indoor sources, we were unable to confirm associations with measured indoor and personal concentrations, as has been the case for previous studies [45,46], (see Additional file 1: Table S1 for the univariate analyses). Moth balls are available with two different formulations in Canada (para-dichlorobenzene or naphthalene) and we did not collect data on which type our study participants used therefore limiting our ability to interpret the influence of this particular source. In a larger study of 288 homes in Michigan, Batterman et al. [8] identified pest repellant use, presence of an attached garage, cigarette smoke, and outdoor sources as contributing to indoor naphthalene concentrations. This may indicate that for many homes, the total naphthalene level reflects a number of smaller sources and cannot easily be attributed to distinct sources without significant statistical power. An exception is the improper use of moth balls, which can be associated with extremely high indoor concentrations of naphthalene [48]. Only 4 homes in the current study reported using moth balls, and they did not have elevated indoor naphthalene concentrations. Health Canada’s Pest Management Regulatory Agency re-evaluated naphthalene pest-control products in 2010 and concluded that they do not present unacceptable risks to human health when used according to label directions. In this study, there were also a limited number of smokers and exposure to SHS was also limited so it was not possible to evaluate the impact of smoking as a source of naphthalene.

We observed that indoor and personal naphthalene concentrations in air were significantly higher at the post-birth visit than the two pre-birth visits. This may be a result of new products being introduced into the home during this period, as well as possible home renovations. Consumer product uses of naphthalene include some commercially available coatings and paints. As we did not specifically ask about these, we are unable to confirm this hypothesis.

Monitoring was quite evenly distributed throughout the year; some seasonality was seen for the indoor air concentrations but not for the biomarkers. In studies in California there was evidence that PAH concentrations increased as temperatures decreased. However, this trend in seasonality was most pronounced for particle phase PAHs. Vapour phase PAHs (i.e., 99% of naphthalene) did not demonstrate any dramatic seasonality [49]. Naphthalene data collected in the Halifax and Edmonton indoor air studies conducted by Health Canada demonstrated that homes sampled during both the winter and summer had similar concentrations to one another [37,43].

**Figure 1:**
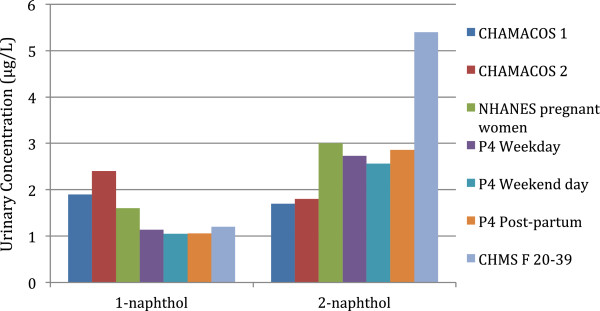
**Median urinary concentrations of 1- and 2-naphthol from the CHAMACOS 1**^**st**^**and 2**^**nd **^**prenatal samples **[39]**, NHANES pregnant women **[39]**, the Canadian Health Measures Survey (CHMS) (females 20 – 39 years of age) **[50]** and our P4 Study.**

The urinary results for this study indicated that there were no significant differences between the three visits with the medians across the 3 sampling periods, ranging from 1.05 to 1.14 for 1-naphthol and 2.56 to 2.86 μg/l for 2-naphthol. The results are similar to those identified by Bouchard et al., [45] where the geometric mean urinary concentrations of 1-naphthol in first morning voids varied from 0.99 to 1.23 μmol/mol creatinine over an 8-month period; the corresponding figures for 2-naphthol were 1.37 to 2.39 μmol/mol creatinine. This indicates that there are some small differences in concentrations by urine collection days which may be lost in our sampling approach of using 24-hour samples in the pregnancy period. Our results for 1-naphthol are also comparable with those from another pregnancy cohort study and with population-based Canadian data, but lower than the Canadian data for 2-naphthol. In the CHAMACOS study of pregnant women in California, the median 1^st^ trimester urinary concentrations of 1- and 2-naphthol were 1.9 and 1.7 μg/L, respectively [39]. For females 20–39 in the Canadian Health Measures Survey (2009–2011), the median 1- and 2-naphthol concentrations in urine (measured by the same laboratory as this study) were 1.2 and 5.4 μg/L, respectively [50], (Figure 1).

Median concentrations of naphthalene in breast milk were significantly lower than observed in previous studies at 7.55 ng/g lipid. The study by Tsang et al. [18] in Hong Kong showed mean naphthalene concentrations of 786 ng/g lipid in breast milk. The researchers found a positive correlation between PAH concentrations in milk and maternal age; our study was unable to reproduce this result. The Turkish study of Çok et al., [19] found that naphthalene was one of the most abundant PAH (contributing 42.6% to the total PAH) identified in human milk from 47 women (mean = 45.75 ng/g lipid). When they separated the analyses by smoking status some of the non-smoking mothers did not have detectable naphthalene concentrations. Similarly, Zanieri et al., [20] found that human milk derived from non-smoking women had approximately half the concentrations of naphthalene compared to smokers (5.56 vs. 10.54 μg/kg of fresh weight milk). The participants in our study were overwhelmingly non-smokers (97%), which may contribute to their low naphthalene concentrations.

Limitations of this study include the relatively small sample size measured and the lack of detailed information on possible indoor sources including the use of deodorizers, sanitizers and air fresheners. Another limitation of the low recruitment rate is the ability to generalise the findings to other pregnant women and populations. Methods for measuring naphthalene in air may be inferior compared to other volatile organic compounds (VOCs) due to methodological limitations (e.g., recovery and reproducibility) [51] although this may be less problematic as the analytical approach used in this study included a naphthalene specific extraction to ensure maximum recovery [37]. In general, very few studies have attempted to identify predictors of naphthalene concentrations in indoor air. The studies that did examine predictors found that indoor naphthalene concentrations were positively and significantly associated with the presence of forced air heating systems with filtration [40], attached garages [40,52], bathroom cleaners/deodorizers [53], and moth control products [9,54].

This study provides valuable information on personal exposure to naphthalene as measured in air, urine and breast milk in the prenatal and post-natal windows. Suspected naphthalene transfer across the placenta has been documented in case studies at high maternal exposure concentrations, with the fetus apparently more vulnerable to naphthalene toxicity than the mother [22,23]. In a pilot study conducted in California, 70% of the amniotic fluids tested positive for 1- or 2-naphthol indicating direct exposure of the young fetus to these phenols; however as the authors did not distinguish between the two metabolites and the study was conducted in an agricultural area, exposure to carbaryl as opposed to naphthalene cannot be ruled out [55]. Since the rate of metabolism of naphthalene in humans is not well characterized, information on the urinary biomarker concentrations may be useful for future attempts to characterize the rate of metabolism in humans in general, or in specific populations (e.g., pregnant women). One possible reason for the lack of association between the biomarkers and the indoor and personal air samples may be the different excretion rates identified by Heikkila et al., [16]. They also found that in workers exposed to occupational concentrations of naphthalene that there were poor associations between air concentrations and the urinary metabolite 1-naphthol with correlation coefficients less than 0.5. Kuusimaki et al., [13] also failed to observe any associations between air samples and urinary metabolites which they attributed to the fact that the workers’ exposures were a combination of diet and occupational exposures. The lack of association between air concentrations and urinary metabolite concentrations could also be due to genetic differences. P450 isoform screening of naphthalene metabolism, performed with human P450 isoforms expressed in baculovirus-infected insect cells, identified CYP1A2 as the most efficient isoform for producing dihydrodiol and 1-naphthol, and CYP3A4 as the most effective for 2-naphthol production [56]. Genetic variations in these enzymes as well as those involved in forming naphthol conjugates (SULT1A1 and UGT1A9) could lead to differences in biotransformation capacity among participants [57,58].

## Conclusions

The results from this study suggest that indoor air monitoring of naphthalene provides a good indication of personal air exposures for pregnant women. Potential sources of biomarker concentrations of naphthalene could not be identified, which is consistent with several other studies. While urinary 1-naphthol and naphthalene in breast milk were associated there were no other associations found for the personal and indoor air concentrations and biomarkers. Potential reasons for this include a potential lack of significant sources, physiological (excretion rates) and behavioural differences that were not captured.

## Abbreviations

BMI: Body mass index; CHMS: Canadian Health Measures Survey; CI: Confidence intervals; INSPQ: Institut national de santé publique du Québec; MDL: Method detection limit; PAH: Polycyclic aromatic hydrocarbon; SHS: Second hand smoke; SG: Specific gravity; TOH: The Ottawa Hospital; VOCs: Volatile organic compounds.

## Competing interests

The authors know of no competing interests either financial or otherwise.

## Authors’ contributions

AJW designed the addition of the naphthalene monitoring, trained staff on monitoring, contributed to the questionnaire design, supervised the naphthalene data management and drafted the manuscript. NAD completed data analysis for the study, and contributed to the interpretation of results and writing of the manuscript. MEH contributed to the design of the addition of the naphthalene monitoring and to the questionnaire and provided input to the manuscript. MF was a co-investigator in the study and contributed to the protocol development of the original study, was involved in study management and implementation, data management and analysis of the naphthalene component and contributed to the writing of the manuscript. LS compiled the naphthalene exposure data and calculated road and highway density variables as well as providing input into the manuscript. CK identified gaps in current knowledge on naphthalene and contributed to the writing of this manuscript. RH contributed to the design of the study, interpretation of naphthalene results and provided input to the manuscript. MW contributed to the design of the study, interpretation of naphthalene results and provided input into the manuscript. TR contributed to the design of the study, interpretation of naphthalene results and provided input into the manuscript. AL, J-FB and ED developed the methods for the naphthalene biomarker analyses and contributed to the manuscript. EG supervised the laboratory biomarker analyses during the study and PB supervised the work associated with the new methods for the biomarker analyses. MF contributed to the design of the study, interpretation of naphthalene results and provided input into the manuscript. PA was a co-investigator in the study and critically revised the manuscript and assisted with the interpretation of the naphthalene results. TEA was the principal investigator of the P4 Study, responsible for the overall design and conduct and contributed to the writing of this manuscript. All authors read and approved the final manuscript.

## Supplementary Material

Additional file 1: Table S1 Univariate models for naphthalene in air and biomarkers. Click here for file

Additional file 2: Figure S1 Weekday (visit T1a) pregnancy personal vs. indoor air naphthalene concentrations. Click here for file
