# Supplementary material for: Urinary and breast milk biomarkers to assess exposure to naphthalene in pregnant women: an investigation of personal and indoor air sources
Source: Environ Health. 2014 Apr 27;13:30. doi: 10.1186/1476-069X-13-30 (PMC4021493; doi:10.1186/1476-069X-13-30)
Supplement: Additional file 1: Table S1 — Univariate models for naphthalene in air and biomarkers. [file 1476-069X-13-30-S1.docx]

| **Pregnancy Indoor Air (logged)** |  |  |  |  |  |  |  |  |
| --- | --- | --- | --- | --- | --- | --- | --- | --- |
| **Full Description** | **Variable** | **Sample** | **R** | **Regression** | **Standard** | **Lower** | **Upper** | ***p*-value** |
|  | **Description** | **Size** | **Square** | **Coefficient** | **Error** | **CI** | **CI** |  |
| Second hand smoke exposure in the home | Second hand smoke exposure inside home | 74 | 0 | -0.151 | 0.409 | -0.953 | 0.652 | 0.714 |
| Road Density surrounding home based on 3-digit postal code | Road Density in 3 digit postal code (km/km2) | 74 | 0 | -0.027 | 0.061 | -0.146 | 0.093 | 0.662 |
| Highway Density surrounding the home based on 3-digit postal code. | Highway Density in 3-digit postal code (km/km2) | 74 | 0 | -0.118 | 0.216 | -0.542 | 0.305 | 0.586 |
| Do you have a garage? | Have a garage (any type) | 52 | 0 | 0.046 | 0.193 | -0.333 | 0.425 | 0.814 |
| Is your garaged attached, with a door connecting it to the home? | Attached garage | 52 | 0.01 | 0.126 | 0.186 | -0.238 | 0.49 | 0.500 |
| Do you have a fireplace or wood stove? | Wood burning fire place | 52 | 0.01 | -0.161 | 0.204 | -0.561 | 0.24 | 0.435 |
| Do you currently have any moth balls? | Moth Balls | 52 | 0.01 | 0.187 | 0.349 | -0.496 | 0.871 | 0.594 |
| In what country were you born? | Born in Canada | 75 | 0.02 |  |  |  |  |  |
|  | no |  |  | 0 |  |  |  |  |
|  | yes |  |  | -0.213 | 0.199 | -0.604 | 0.178 | 0.289 |
| Season of sampling | Season | 62 | 0.14 |  |  |  |  |  |
|  | fall |  |  | 0.219 | 0.228 | -0.228 | 0.666 | 0.341 |
|  | spring |  |  | 0.087 | 0.25 | -0.403 | 0.578 | 0.728 |
|  | summer |  |  | 0 |  |  |  |  |
|  | winter |  |  | -0.457 | 0.241 | -0.929 | 0.015 | 0.063* |
| * signficant at the 0.10 level; ** signficant at the 0.05 level | |  |  |  |  |  |  |  |
|  |  |  |  |  |  |  |  |  |
| **Post-Partum Indoor Air (logged)** |  |  |  |  |  |  |  |  |
| **Full Description** | **Variable** | **Sample** | **R** | **Regression** | **Standard** | **Lower** | **Upper** | ***p*-value** |
|  | **Description** | **Size** | **Square** | **Coefficient** | **Error** | **CI** | **CI** |  |
| Second hand smoke exposure in the home | Second hand smoke exposure inside home | 59 | 0.01 | 0.473 | 0.593 | -0.69 | 1.636 | 0.429 |
| Road Density surrounding home based on 3-digit postal code | Road Density in 3 digit postal code (km/km2) | 50 | 0.03 | -0.08 | 0.066 | -0.21 | 0.05 | 0.235 |
| Highway Density surrounding the home based on 3-digit postal code. | Highway Density in 3-digit postal code (km/km2) | 50 | 0.01 | -0.154 | 0.249 | -0.641 | 0.333 | 0.539 |
| Do you have a garage? | Garage (any type) | 41 | 0 | -0.061 | 0.161 | -0.377 | 0.255 | 0.706 |
| Is your garaged attached, with a door connecting it to the home? | Attached garage | 41 | 0.03 | 0.152 | 0.151 | -0.145 | 0.449 | 0.322 |
| Do you have a fireplace or wood stove? | Wood burning fire place | 41 | 0.1 | -0.335 | 0.164 | -0.657 | -0.014 | 0.048* |
| In what country were you born? | Born in Canada | 60 | 0.03 |  |  |  |  |  |
|  | no |  |  | 0 |  |  |  |  |
|  | yes |  |  | 0.249 | 0.191 | -0.125 | 0.624 | 0.198 |
| Season of sampling | Season | 58 | 0.14 |  |  |  |  |  |
|  | fall |  |  | -0.793 | 0.276 | -1.33 | -0.252 | 0.006** |
|  | spring |  |  | -0.427 | 0.235 | -0.887 | 0.033 | 0.074 |
|  | summer |  |  | 0 |  |  |  |  |
|  | winter |  |  | -0.529 | 0.239 | -0.998 | -0.06 | 0.031** |
| * signficant at the 0.10 level; ** signficant at the 0.05 level | |  |  |  |  |  |  |  |
|  |  |  |  |  |  |  |  |  |
| **Pregnancy Personal Air Monitors (logged)** | |  |  |  |  |  |  |  |
| **Full Variable Name** | **Variable description** | **Sample** | **R** | **Regression** | **Standard** | **Lower** | **Upper** |  |
|  |  | **Size** | **Square** | **Coefficient** | **Error** | **CI** | **CI** | ***p*-value** |
| Second hand smoke exposure in home, vehicle or workplace? | Second Hand Smoke | 74 | 0.01 | 0.188 | 0.233 | -0.268 | 0.645 | 0.422 |
| Does anyone smoke inside your home every day or almost every day? | Second Hand Smoke in home | 73 | 0 | 0.021 | 0.488 | -0.936 | 0.979 | 0.966 |
| Road Density surrounding home based on 3-digit postal code | Road Density in 3 digit postal code (km/km2) | 72 | 0 | 0.03 | 0.074 | -0.114 | 0.175 | 0.682 |
| Highway Density surrounding the home based on 3-digit postal code. | Highway Density in 3-digit postal code (km/km2) | 72 | 0 | 0.092 | 0.261 | -0.421 | 0.604 | 0.727 |
| Do you have a garage? | Garage (any type) | 51 | 0.01 | 0.211 | 0.246 | -0.272 | 0.694 | 0.396 |
| Is your garaged attached, with a door connecting it to the home? | Attached garage | 51 | 0 | 0.052 | 0.237 | -0.413 | 0.517 | 0.827 |
| Do you have a fireplace or wood stove? | Wood burning fire place | 51 | 0.04 | -0.349 | 0.261 | -0.861 | 0.163 | 0.188 |
| Do you currently have any moth balls? | Moth Balls | 51 | 0.01 | 0.338 | 0.439 | -0.522 | 1.198 | 0.444 |
| Measured Indoor Naphthalene levels | Indoor Naphthalene levels | 73 | 0.6 | 0.675 | 0.066 | 0.546 | 0.804 | 0** |
| In what country were you born? | Born in Canada | 74 | 0.01 |  |  |  |  |  |
|  | no |  |  | 0 |  |  |  |  |
|  | yes |  |  | -0.24 | 0.238 | -0.706 | 0.227 | 0.318 |
| Season of sampling | Season | 61 | 0.27 |  |  |  |  |  |
|  | fall |  |  | 0.142 | 0.256 | -0.361 | 0.644 | 0.583 |
|  | spring |  |  | -0.112 | 0.284 | -0.669 | 0.445 | 0.695 |
|  | summer |  |  | 0 |  |  |  |  |
|  | winter |  |  | -1.02 | 0.284 | -1.58 | -0.466 | 0.001** |
| * signficant at the 0.10 level; ** signficant at the 0.05 level | |  |  |  |  |  |  |  |
|  |  |  |  |  |  |  |  |  |
| **Post-Partum Personal Air Monitors (logged)** | |  |  |  |  |  |  |  |
| **Full Variable Name** | **Variable Description** | **Sample** | **R** | **Regression** | **Standard** | **Lower** | **Upper** | ***p*-value** |
|  |  | **Size** | **Square** | **Coefficient** | **Error** | **CI** | **CI** |  |
| Second hand smoke exposure in home, vehicle or workplace? | Second Hand Smoke | 61 | 0 | 0.096 | 0.189 | -0.274 | 0.467 | 0.612 |
| Does anyone smoke inside your home every day or almost every day? | Second Hand Smoke in home | 60 | 0 | -0.237 | 0.623 | -1.46 | 0.984 | 0.705 |
| Road Density surrounding home based on 3-digit postal code | Road Density in 3 digit postal code (km/km2) | 51 | 0.03 | -0.083 | 0.068 | -0.216 | 0.051 | 0.23 |
| Highway Density surrounding the home based on 3-digit postal code. | Highway Density in 3-digit postal code (km/km2) | 51 | 0.02 | -0.223 | 0.251 | -0.716 | 0.269 | 0.378 |
| Do you have a garage? | Garage (any type) | 42 | 0.01 | -0.124 | 0.188 | -0.492 | 0.243 | 0.512 |
| Is your garaged attached, with a door connecting it to the home? | Attached garage | 42 | 0 | 0.054 | 0.178 | -0.294 | 0.403 | 0.762 |
| Do you have a fireplace or wood stove? | Wood burning fire place | 42 | 0.04 | -0.252 | 0.198 | -0.641 | 0.137 | 0.211 |
| Measured Indoor Napthalene levels | Indoor Napthalene levels | 60 | 0.6 | 0.282 | 0.03 | 0.222 | 0.342 | 0** |
| In what country were you born? | Born in Canada | 61 | 0.01 |  |  |  |  |  |
|  | no |  |  | 0 |  |  |  |  |
|  | yes |  |  | 0.188 | 0.199 | -0.202 | 0.577 | 0.348 |
| Season of sampling | Season | 59 | 0.16 |  |  |  |  |  |
|  | fall |  |  | -0.894 | 0.28 | -1.44 | -0.346 | 0.002** |
|  | spring |  |  | -0.527 | 0.238 | -0.993 | -0.061 | 0.031** |
|  | summer |  |  | 0 |  |  |  |  |
|  | winter |  |  | -0.606 | 0.241 | -1.08 | -0.134 | 0.015** |
| * signficant at the 0.10 level; ** signficant at the 0.05 level | |  |  |  |  |  |  |  |
|  |  |  |  |  |  |  |  |  |
| **Pregnancy Maternal Pooled Urine 1-napthol Levels (logged)** | |  |  |  |  |  |  |  |
|  | **Variable** | **Sample** | **R** | **Regression** | **Standard** | **Lower** | **Upper** | ***p*-value** |
|  | **Description** | **Size** | **Square** | **Coefficient** | **Error** | **CI** | **CI** |  |
| Maternal Age at recruitment | maternal age | 79 | 0.04 | 0.034 | 0.02 | -0.004 | 0.073 | 0.086 |
| Maternal pre-pregnancy body mass index (BMI) | maternal preg pregnancy BMI | 71 | 0 | 0.008 | 0.023 | -0.037 | 0.053 | 0.728 |
| Second hand smoke exposure in home, vehicle or workplace? | Second Hand Smoke inside home | 79 | 0 | -0.207 | 0.52 | -1.23 | 0.812 | 0.692 |
| Does anyone smoke inside your home every day or almost every day? | Second Hand Smoke | 80 | 0 | -0.071 | 0.235 | -0.532 | 0.39 | 0.764 |
| Have you eer smoked at least 100 cigarettes over you lifetime? | Ever smoked 100 cigarrettes | 78 | 0.05 | 0.41 | 0.211 | -0.003 | 0.823 | 0.055* |
| Road Density surrounding home based on 3-digit postal code | Road Density in 3 digit postal code (km/km2) | 78 | 0.01 | 0.047 | 0.076 | -0.102 | 0.197 | 0.536 |
| Highway Density surrounding the home based on 3-digit postal code. | Highway Density in 3-digit postal code (km/km2) | 78 | 0 | -0.046 | 0.271 | -0.577 | 0.485 | 0.865 |
| Do you have a garage? | Garage (any type) | 56 | 0.02 | -0.24 | 0.264 | -0.757 | 0.277 | 0.367 |
| Is your garaged attached, with a door connecting it to the home? | Attached Garage | 56 | 0.01 | -0.178 | 0.26 | -0.687 | 0.332 | 0.497 |
| Do you have a fireplace or wood stove? | Wood burning Fire place | 56 | 0 | -0.05 | 0.287 | -0.613 | 0.513 | 0.862 |
| Do you currently have any moth balls? | Moth Balls | 56 | 0 | 0.151 | 0.455 | -0.74 | 1.042 | 0.741 |
| Measured Pregnancy Indoor Naphthalene levels (logged for analysis) | Indoor Naphthelene levels | 75 | 0.03 | 0.22 | 0.146 | -0.067 | 0.507 | 0.138 |
| Measured Pregnancy Personal Naphthalene levels (logged for analysis) | Personal Naphthalene levels | 74 | 0.01 | 0.12 | 0.125 | -0.126 | 0.365 | 0.342 |
| In what country were you born? | Born in Canada | 80 | 0.01 |  |  |  |  |  |
|  | no |  |  | 0 |  |  |  |  |
|  | yes |  |  | -0.213 | 0.239 | -0.681 | 0.256 | 0.377 |
| Season of sampling | Season | 64 | 0.08 |  |  |  |  |  |
|  | fall |  |  | 0.173 | 0.288 | -0.391 | 0.737 | 0.550 |
|  | spring |  |  | 0.68 | 0.319 | 0.054 | 1.305 | 0.037** |
|  | summer |  |  | 0 |  |  |  |  |
|  | winter |  |  | 0.084 | 0.303 | -0.509 | 0.678 | 0.782 |
| * signficant at the 0.10 level; ** signficant at the 0.05 level | |  |  |  |  |  |  |  |
|  |  |  |  |  |  |  |  |  |
| **Pregnancy Maternal Pooled Urine 2-napthol Levels (logged)** | |  |  |  |  |  |  |  |
| **Full Variable Description** | **Variable** | **Sample** | **R** | **Regression** | **Standard** | **Lower** | **Upper** | ***p*-value** |
|  | **Description** | **Size** | **Square** | **Coefficient** | **Error** | **CI** | **CI** |  |
| Maternal Age at recruitment | maternal age | 79 | 0.02 | -0.019 | 0.017 | -0.053 | 0.015 | 0.282 |
| Maternal pre-pregnancy body mass index (BMI) | maternal preg pregnancy BMI | 71 | 0.06 | 0.039 | 0.019 | 0.002 | 0.077 | 0.043** |
| Second hand smoke exposure in home, vehicle or workplace? | Second Hand Smoke | 79 | 0 | 0.154 | 0.449 | -0.727 | 1.034 | 0.733 |
| Does anyone smoke inside your home every day or almost every day? | Second Hand Smoke in home | 80 | 0.02 | -0.225 | 0.203 | -0.623 | 0.173 | 0.271 |
| Have you eer smoked at least 100 cigarettes over you lifetime? | Ever smoked 100 cigarrettes | 78 | 0 | 0.017 | 0.186 | -0.348 | 0.382 | 0.927 |
| Road Density surrounding home based on 3-digit postal code | Road Density in 3 digit postal code (km/km2) | 78 | 0.02 | -0.071 | 0.065 | -0.199 | 0.057 | 0.281 |
| Highway Density surrounding the home based on 3-digit postal code. | Highway Density in 3-digit postal code (km/km2) | 78 | 0.02 | -0.283 | 0.231 | -0.735 | 0.169 | 0.224 |
| Do you have a garage? | Garage (any type) | 56 | 0 | -0.103 | 0.198 | -0.492 | 0.286 | 0.605 |
| Is your garaged attached, with a door connecting it to the home? | Attached Garage | 56 | 0.01 | -0.128 | 0.195 | -0.509 | 0.253 | 0.514 |
| Do you have a fireplace or wood stove? | Wood burning Fire place | 56 | 0.01 | -0.185 | 0.214 | -0.604 | 0.234 | 0.390 |
| Do you currently have any moth balls? | Moth Balls | 56 | 0 | -0.159 | 0.34 | -0.825 | 0.508 | 0.642 |
| Measured Pregnancy Indoor Naphthalene levels (logged for analysis) | Indoor Naphthelene levels | 75 | 0.01 | -0.121 | 0.127 | -0.371 | 0.128 | 0.343 |
| Measured Pregnancy Personal Naphthalene levels (logged for analysis) | Personal Naphthalene leves | 74 | 0.03 | -0.158 | 0.106 | -0.366 | 0.049 | 0.140 |
| In what country were you born? | Born in Canada | 80 | 0.04 |  |  |  |  |  |
|  | no |  |  | 0 |  |  |  |  |
|  | yes |  |  | 0.387 | 0.204 | -0.013 | 0.787 | 0.062 |
| Season of sampling | Season | 64 | 0.13 |  |  |  |  |  |
|  | fall |  |  | 0.462 | 0.23 | 0.011 | 0.913 | 0.049** |
|  | spring |  |  | 0.006 | 0.255 | -0.494 | 0.506 | 0.981 |
|  | summer |  |  | 0 |  |  |  |  |
|  | winter |  |  | 0.546 | 0.242 | 0.072 | 1.02 | 0.028** |
| * signficant at the 0.10 level; ** signficant at the 0.05 level | |  |  |  |  |  |  |  |
|  |  |  |  |  |  |  |  |  |
|  |  |  |  |  |  |  |  |  |
| **Post-Partum Maternal Pooled Urine 1-napthol Levels (logged)** | |  |  |  |  |  |  |  |
| **Full Variable Description** | **Variable** | **Sample** | **R** | **Regression** | **Standard** | **Lower** | **Upper** | ***p*-value** |
|  | **Description** | **Size** | **Square** | **Coefficient** | **Error** | **CI** | **CI** |  |
| Maternal Age at recruitment | maternal age | 61 | 0 | 0.011 | 0.028 | -0.045 | 0.066 | 0.703 |
| Maternal pre-pregnancy body mass index (BMI) | maternal pre pregnancy BMI | 58 | 0.04 | 0.044 | 0.029 | -0.012 | 0.1 | 0.133 |
| Does anyone smoke inside your home every day or almost every day? | Second Hand Smoke inside home | 61 | 0 | -0.398 | 0.997 | -2.35 | 1.557 | 0.692 |
| Second hand smoke exposure in home, vehicle or workplace? | Second Hand Smoke | 62 | 0.01 | -0.182 | 0.296 | -0.762 | 0.398 | 0.541 |
| Have you eer smoked at least 100 cigarettes over you lifetime? | Ever smoked 100 cigarrettes | 60 | 0.04 | 0.434 | 0.275 | -0.105 | 0.973 | 0.120 |
| Road Density surrounding home based on 3-digit postal code | Road Density in 3 digit postal code (km/km2) | 52 | 0.04 | -0.161 | 0.107 | -0.37 | 0.048 | 0.138 |
| Highway Density surrounding the home based on 3-digit postal code. | Highway Density in 3-digit postal code (km/km2) | 52 | 0.14 | -1.08 | 0.378 | -1.82 | -0.337 | 0.006** |
| Do you have a garage? | Garage (any type) | 42 | 0.06 | -0.451 | 0.292 | -1.02 | 0.12 | 0.13 |
| Is your garaged attached, with a door connecting it to the home? | Attached Garage | 42 | 0.01 | -0.199 | 0.276 | -0.74 | 0.342 | 0.475 |
| Do you have a fireplace or wood stove? | Wood burning Fire place | 42 | 0.05 | -0.464 | 0.317 | -1.09 | 0.158 | 0.151 |
| Do you currently have any moth balls? | Moth Balls | 42 | 0 | 0 |  |  |  |  |
| Measured Pregnancy Indoor Naphthalene levels (logged for analysis) | Indoor Naphthelene levels | 57 | 0 | -0.095 | 0.2 | -0.488 | 0.298 | 0.639 |
| Measured Pregnancy Personal Naphthalene levels (logged for analysis) | Personal Naphthalene leves | 57 | 0.01 | -0.096 | 0.163 | -0.414 | 0.223 | 0.559 |
| In what country were you born? | Born in Canada | 62 | 0.01 |  |  |  |  |  |
|  | no |  |  | 0 |  |  |  |  |
|  | yes |  |  | 0.202 | 0.311 | -0.408 | 0.813 | 0.518 |
| Season of sampling | Season at T1 | 61 | 0.05 |  |  |  |  |  |
|  | fall |  |  | 0.346 | 0.475 | -0.584 | 1.277 | 0.469 |
|  | spring |  |  | -0.187 | 0.413 | -0.997 | 0.623 | 0.652 |
|  | summer |  |  | 0 |  |  |  |  |
|  | winter |  |  | 0.255 | 0.416 | -0.56 | 1.07 | 0.542 |
| * signficant at the 0.10 level; ** signficant at the 0.05 level | |  |  |  |  |  |  |  |
|  |  |  |  |  |  |  |  |  |
| **Post-Partum Maternal Pooled Urine 2-napthol Levels (logged)** | |  |  |  |  |  |  |  |
| **Full Variable Description** | **Variable** | **Sample** | **R** | **Regression** | **Standard** | **Lower** | **Upper** | ***p*-value** |
|  | **Description** | **Size** | **Square** | **Coefficient** | **Error** | **CI** | **CI** |  |
| Maternal Age at recruitment | maternal age | 61 | 0.02 | -0.032 | 0.03 | -0.09 | 0.026 | 0.283 |
| Maternal pre-pregnancy body mass index (BMI) | maternal preg pregnancy BMI | 58 | 0.03 | 0.038 | 0.03 | -0.021 | 0.096 | 0.209 |
| Does anyone smoke inside your home every day or almost every day? | Second Hand Smoke inside home | 61 | 0.08 | 2.217 | 1.011 | 0.235 | 4.199 | 0.032 |
| Second hand smoke exposure in home, vehicle or workplace? | Second Hand Smoke | 62 | 0.03 | -0.447 | 0.308 | -1.05 | 0.157 | 0.152 |
| Have you ever smoked at least 100 cigarettes over you lifetime? | Ever smoked 100 cigarrettes | 60 | 0 | -0.058 | 0.292 | -0.631 | 0.515 | 0.844 |
| Road Density surrounding home based on 3-digit postal code | Road Density in 3 digit postal code (km/km2) | 52 | 0.05 | -0.178 | 0.109 | -0.392 | 0.036 | 0.109 |
| Highway Density surrounding the home based on 3-digit postal code. | Highway Density in 3-digit postal code (km/km2) | 52 | 0.09 | -0.906 | 0.398 | -1.69 | -0.126 | 0.027** |
| Do you have a garage? | Garage (any type) | 42 | 0.04 | -0.41 | 0.332 | -1.06 | 0.241 | 0.224 |
| Is your garaged attached, with a door connecting it to the home? | Attached Garage | 42 | 0 | -0.099 | 0.313 | -0.713 | 0.515 | 0.753 |
| Do you have a fireplace or wood stove? | Wood burning Fire place | 42 | 0 | -0.104 | 0.367 | -0.824 | 0.616 | 0.778 |
| Measured Post-Partum Indoor Naphthalene levels (logged for analysis) | Indoor Naphthelene levels | 57 | 0.02 | -0.21 | 0.194 | -0.591 | 0.171 | 0.285 |
| Measured Post-Partum Personal Naphthalene levels (logged for analysis) | Personal Naphthalene levels | 57 | 0.02 | -0.174 | 0.162 | -0.492 | 0.145 | 0.289 |
| In what country were you born? | Born in Canada | 62 | 0.03 |  |  |  |  |  |
|  | no |  |  | 0 |  |  |  |  |
|  | yes |  |  | 0.413 | 0.325 | -0.224 | 1.051 | 0.208 |
| Season of sampling | Season | 61 | 0.04 |  |  |  |  |  |
|  | fall |  |  | 0.155 | 0.504 | -0.833 | 1.144 | 0.759 |
|  | spring |  |  | 0.151 | 0.439 | -0.709 | 1.012 | 0.732 |
|  | summer |  |  | 0 |  |  |  |  |
|  | winter |  |  | 0.522 | 0.442 | -0.344 | 1.388 | 0.242 |
| * signficant at the 0.10 level; ** signficant at the 0.05 level | |  |  |  |  |  |  |  |
|  |  |  |  |  |  |  |  |  |
| **Lipid Adjusted Napthalene in Breast Milk (logged)** | |  |  |  |  |  |  |  |
| **Full Variable Description** | **Variable** | **Sample** | **R** | **Regression** | **Standard** | **Lower** | **Upper** | ***p*-value** |
|  | **Description** | **Size** | **Square** | **Coefficient** | **Error** | **CI** | **CI** |  |
| Maternal Age at recruitment | maternal age | 51 | 0 | 0.004 | 0.022 | -0.039 | 0.046 | 0.872 |
| Maternal pre-pregnancy body mass index (BMI) | maternal preg pregnancy BMI | 48 | 0 | 0.007 | 0.022 | -0.036 | 0.05 | 0.762 |
| Does anyone smoke inside your home every day or almost every day? | Second Hand Smoke inside home | 51 | 0 | 0 |  |  |  |  |
| Second hand smoke exposure in home, vehicle or workplace? | Second Hand Smoke | 52 | 0.02 | -0.22 | 0.215 | -0.641 | 0.202 | 0.312 |
| Have you eer smoked at least 100 cigarettes over you lifetime? | Ever smoked 100 cigarrettes | 50 | 0 | 0.099 | 0.214 | -0.32 | 0.518 | 0.645 |
| Road Density surrounding home based on 3-digit postal code | Road Density in 3 digit postal code (km/km2) | 46 | 0.05 | -0.106 | 0.069 | -0.242 | 0.029 | 0.132 |
| Highway Density surrounding the home based on 3-digit postal code. | Highway Density in 3-digit postal code (km/km2) | 46 | 0.02 | -0.226 | 0.268 | -0.752 | 0.299 | 0.403 |
| Do you have a garage? | Garage (any type) | 34 | 0.03 | -0.255 | 0.266 | -0.777 | 0.267 | 0.345 |
| Is your garaged attached, with a door connecting it to the home? | Attached Garage | 34 | 0 | 0.041 | 0.246 | -0.441 | 0.523 | 0.868 |
| Do you have a fireplace or wood stove? | Wood burning Fire place | 34 | 0.02 | -0.212 | 0.288 | -0.776 | 0.352 | 0.467 |
| Measured Post-Partum Indoor Naphthalene levels (logged for analysis) | Indoor Naphthelene levels | 49 | 0.14 | 0.345 | 0.127 | 0.096 | 0.594 | 0.009** |
| Measured Post-Partum Personal Naphthalene levels (logged for analysis) | Personal Naphthalene levels | 50 | 0.16 | 0.357 | 0.118 | 0.125 | 0.588 | 0.004** |
| Measured 1-naphthol levels in maternal urine post-partum (logged for analysis) | 1-naphthol levels post-partum | 50 | 0.15 | 0.249 | 0.086 | 0.081 | 0.417 | 0.005** |
| Measured 2-naphthol levels in maternal urine post-partum (logged for analysis) | 2-naphthol levels post-partum | 50 | 0.04 | 0.128 | 0.093 | -0.054 | 0.311 | 0.174 |
| In what country were you born? | Born in Canada | 52 | 0.01 |  |  |  |  |  |
|  | no |  |  | 0 |  |  |  |  |
|  | yes |  |  | 0.15 | 0.231 | -0.304 | 0.604 | 0.520 |
| Season of sampling | Season | 50 | 0.04 |  |  |  |  |  |
|  | fall |  |  | 0.315 | 0.318 | -0.307 | 0.938 | 0.326 |
|  | spring |  |  | -0.037 | 0.286 | -0.597 | 0.523 | 0.897 |
|  | summer |  |  | 0 |  |  |  |  |
|  | winter |  |  | 0.067 | 0.281 | -0.483 | 0.618 | 0.812 |
| * significant at the 0.10 level; ** significant at the 0.05 level | |  |  |  |  |  |  |  |
