# Supplementary figures and images for: Urinary and breast milk biomarkers to assess exposure to naphthalene in pregnant women: an investigation of personal and indoor air sources
Source: Environ Health. 2014 Apr 27;13:30. doi: 10.1186/1476-069X-13-30 (PMC4021493; doi:10.1186/1476-069X-13-30)

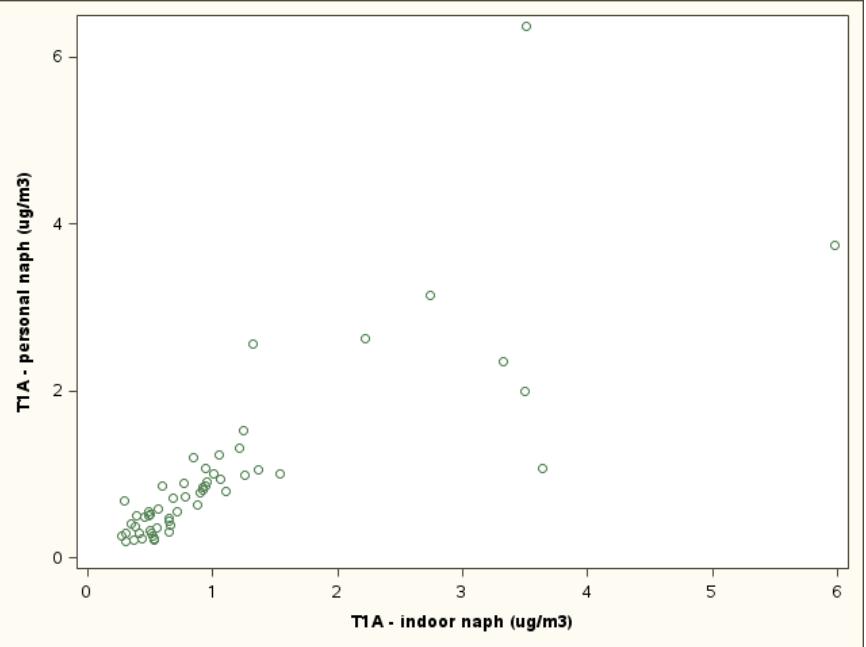

Supplement: Additional file 2: Figure S1 — Weekday (visit T1a) pregnancy personal vs. indoor air naphthalene concentrations. [file 1476-069X-13-30-S2.pdf]
